# Effects of different combinations of antibacterial compound supplements in calf pellets on growth performance, health, blood parameters, and rumen microbiome of dairy calves

**DOI:** 10.3389/fvets.2024.1376758

**Published:** 2024-05-13

**Authors:** Shujie Li, Changjian Wang, Hanfang Zeng, Zhaoyu Han

**Affiliations:** College of Animal Science and Technology, Nanjing Agricultural University, Nanjing, China

**Keywords:** attapulgite, plant essential oil, chitosan oligosaccharide, growth performance, rumen microbiome, calf

## Abstract

This study investigated the effects of different combinations of antibacterial compounds (attapulgite, plant essential oils, and chitosan oligosaccharides) on growth performance, blood biochemical parameters, and rumen microbiome of calves. A total of 48 preweaning calves were randomly divided into four groups (*n* = 12 per group), and fed the following full mixed-ration granule diets for the 67-d-feeding trial: (1) basal diet (control group); (2) basal diet +1,000 g/t attapulgite, plant essential oils, and chitosan oligosaccharide (AEOCO group); (3) basal diet +1,000 g/t attapulgite and chitosan oligosaccharide (ACO group); and (4) basal diet +1,000 g/t attapulgite and plant essential oil (AEO group). The results showed that the daily weight gain of the AEOCO and AEO groups significantly increased (*p* < 0.05), whereas the feed conversion ratio decreased compared with that of the control group. Among the three treatment groups, AEO group showed the most positive effect, with the diarrhea rate reduced by 68.2% compared with that of the control group. Total protein and globulin levels were lower in the AEO group than in the control group. Albumin levels were higher in the AEOCO and AEO groups than in the control group. Immunoglobulin A, immunoglobulin G, and immunoglobulin M concentrations were higher in the AEOCO group (*p* < 0.05) than in the control group. The interleukin-6 concentration was lower in the AEOCO and AEO groups than in the control group (*p* < 0.05). The Chao 1 richness and ACE indices were higher in the AEOCO group than in the control group (*p* < 0.05). The ACO group had a significantly lower (*p* < 0.05) relative abundance of *Firmicutes* than the control group. The relative abundance of *Bacteroidetes* was the lowest in the control group, whereas that of *Spirochaetota* and *Fibrobacteriota* was the highest (*p* < 0.05). The relative abundance of *Succiniclasticum* was higher in the ACO and AEO groups (*p* < 0.05). These findings indicate that the combination of attapulgite, plant essential oils, and chitosan oligosaccharides has ameliorative effects on the growth performance, blood parameters, and rumen microbiome of calves.

## 1 Introduction

Diarrhea is common when feeding calves, with severe diarrhea resulting in calf death and economic losses (1, 2). The incidence of diarrhea in calves on dairy farms ranges from 20 to 50%, with a mortality rate of 20–40%. Currently, antibiotics are the most common treatment for calf diarrhea (3, 4). Published research shows that the addition of antibiotics to calf diets can reduce calf diarrhea and improve calf survival rates (5–7). However, problems, such as drug resistance and drug residues, remain (8). Antibiotic abuse can lead to digestive tract microbiome disorders in calves. Tetracyclines may also significantly affect rumen microbes in calves (9). Calves are usually fed pellets during the weaning process to supplement nutrients required for growth. Hay-based Total Mixed Rations (TMR) may also be fed to calves during the weaning process (10). New methods of treatment are required to prevent and control calf diarrhea and reduce the dependence of the breeding industry on antibiotics. Functional additives, such as attapulgite, plant essential oils, and chitosan oligosaccharides are possible alternatives to antibacterial additives.

Attapulgite is a magnesium-aluminum silicate mineral with a rod crystal morphology and pore structure (11). It is used as a feed additive owing to its high adsorption capacity (12). It can absorb harmful substances such as intestinal pathogenic bacteria and mycotoxins, and plays a key role in the prevention and treatment of diarrhea and gastrointestinal diseases in animals (13). The supplementation of piglet diets with attapulgite can improve the intestinal barrier function of weanling piglets and substitute for high doses of ZnO and antibiotics (13). In addition, the addition of 2000 mg/kg PAL to feed can increase the ADG of Holstein calves, improve calf immune levels and antioxidant capacity, regulate intestinal flora, and reduce the incidence of diarrhea (14). Plant essential oils are natural and non-drug-resistant, and can improve the growth performance of livestock by preventing intestinal inflammation, regulating intestinal microflora, and inhibiting the growth of harmful bacteria (15–18). Plant essential oils can alleviate diarrhea in piglets by increasing the digestive enzyme activity (17). Nannoni et al. (19) found that Lavender Essential Oil Inhalation reduced tail lesion severity in Italian heavy pigs (19). Plant essential oils can also increase feed intake and nutrient transport in growing sheep (20). Dietary plant essential oils can also increased the yield of energy-corrected milk and prevented body weight loss after parturition (21). Chitosan oligosaccharides have antibacterial and bacteriostatic effects and can effectively inhibit the reproduction of harmful microorganisms. They also significantly promote animal growth. The addition of chitosan oligosaccharides to the diet of weaned piglets can promote intestinal development, increase apparent digestibility, and reduce diarrhea rates (22, 23). Adding 0.07% chitosan oligosaccharide to the diet of weaned piglets increased their average daily feed intake and weight gain (24). The addition of 600 mg of chitosan to the basal diet has reduced the incidence of diarrhea in calves by 62.9% (25).

However, the combined use of these three new additives (attapulgite, plant essential oils, and chitosan oligosaccharides) is currently under-researched. We hypothesized that a combination of additives would be more effective for the growth, health, and rumen microbiome of calves. Therefore, we explored the effects of different combinations of antibacterial additives on the growth, health, and rumen microbiome of calves, with the aim of providing a new experimental basis for the application of antibacterial materials in dairy farming.

## 2 Materials and methods

The experimental design and procedures were performed in accordance with the Institutional Animal Care and Use Committee of the Nanjing Agricultural University, China (SYXK2011-0036).

### 2.1 Antibacterial compound

Three antibacterial compounds (attapulgite, plant essential oils, and chitosan oligosaccharides) were divided into different combinations. AEOCO group: attapulgite, plant essential oils, and chitosan oligosaccharide; ACO group: attapulgite and chitosan oligosaccharide; AEO group: attapulgite and plant essential oils. Each antibacterial material combination used attapulgite as the carrier; the addition of plant essential oil was 10 g/1000 g, and the addition of chitosan oligosaccharide was 50 g/1000 g. The essential oil used was oregano vulgare. All combinations were mixed with calf pellets and processed into granules for supplementary feeding prior to weaning.

### 2.2 Animal experimental design

A total of 48 Holstein calves based on age and weight (birth weight = 39.2 ± 2.16 kg; mean ± SD; China) were selected and randomly divided into 4 groups. The average age was 7 days and the age difference between the calves was within 2 days. The calves in the four treatment groups were fed the following full mixed-ration granule diets for the 67-d-feeding trial: (1) control group: basal diet; (2) AEOCO group: basal diet +1,000 g/t attapulgite, plant essential oils, and chitosan oligosaccharide; (3) ACO group: basal diet +1,000 g/t attapulgite and chitosan oligosaccharide; and (4) AEO group: basal diet +1,000 g/t attapulgite and plant essential oil. The experiment was performed at the commercial Jie Long Farm in Huai, China. The composition of the ingredients and nutrient levels are listed in Table 1. Calves were raised in single stalls on calf islands and fed milk and water separately. Crude protein, starch, ash, minerals, acid detergent fibers, and neutral detergent fibers were determined according to previously described methods of previous study (26–28).

**Table 1:** Composition and nutrient levels of the basal diet (dry matter (DM) basis) %.

Ingredients	Content, % of DM
Corn	40.32
Soybean meal	34.86
Wheatbran	2.94
Cottonseed meal	6.80
Molasses	4.42
Wheat middlings	7.79
CaCO_3_	1.66
Soybean oil	0.78
NaCl	0.10
CaHPO_4_	0.10
MgO	0.06
Premix	0.17
Chemical composition, % of DM	
DM	86.77
CP	24.66
NDF	9.82
ADF	4.61
EE	4.48
Ash	3.60
Ca	0.79
P	0.51

The premix provided the following per kg of the diet: VA 9000 IU, VD 5000 IU, VE 47.22 mg, Fe 80 mg, Cu 12 mg, Zn 108.81 mg, Mn 79.99 mg, Se 15 mg, Co 0.36 mg.

Calves were milk-fed according to the farm plan and received 6 L/d of milk divided into two feedings (06, 00 and 16:00). The milk replacer powder was diluted in warm water at 37°C in a ratio of 6:1 (6 L of water to 1 kg of milk replacer). The main ingredients of the milk replacement powder were lactose (39%), protein (22%), fat (20%), moisture (5.0%), and coarse ash (9.0%). The trial lasted 67 days.

### 2.3 Assessment of growth performance and diarrhea of calves

Milk consumption and calf pellet feed intake of each group of calves were recorded daily, and the average daily feed intake was calculated. Each calf was weighed once a month and the average daily weight gain was calculated. The calf fecal score was evaluated 2 days per week. A calf fecal scoring system was used at the farm. The standards were as follows: 5 = very firm, 4 = firm, 3 = viscous, 2 = very loose, and 1 = watery. The number of calves with and without diarrhea was recorded, and the diarrhea rate was calculated. Diarrhea incidence (%) = [number of calves with diarrhea/ (number of test calves × test days)] × 100.

### 2.4 Blood sample collection and measurement

At the end of the experiment, 10 mL of blood was collected from the tail vein of each calf and the serum was separated and stored at −20°C. The following indicators were determined: serum protein contents: total protein (TP), albumin (ALB), globulin (GLB); serum immune contents: immunoglobulin M (IgM), immunoglobulin A (IgA), immunoglobulin G (IgG), tumor necrosis factor (TNF-α), interleukin-6 (IL-6), and interferon (IFN-γ); antioxidant contents: glutathione peroxidase (GSH-PX), superoxide dismutase (SOD), malondialdehyde (MDA), and total antioxidant capacity (T-AOC). The kit was purchased from Nanjing Aoqing Biotechnology. Serum biochemical indicators were determined using a biochemical analyzer (Chemray 800, Rayto, Shenzhen, China) and a microplate reader (Tecan, F50, China).

### 2.5 Rumen sample collection and measurement

At the end of the experiment, three calves from each treatment group were sampled using a rumen fluid collector to evaluate rumen microbiome and fermentation parameters. Immediately after collection of rumen fluid, the freshly collected rumen fluid was filtered through four layers of gauze, and its pH was measured. The remaining rumen fluid samples were dispensed into centrifuge tubes and frozen at −80°C for determination of rumen fermentation parameters and microbial flora.

### 2.6 Microbiome sequencing and bioinformatics analysis

A DNA Kit (D4015, Omega, USA) was used to extract total genomic DNA from rumen fluid according to the manufacturer’s protocols. For bacterial diversity analysis, DNA samples from the rumen fluid were used as templates. The V4-V5 variable regions of 16S rRNA genes were amplified by PCR with primers 515F 5′-barcode- GTGCCAGCMGCCGCGG)-3′ and 907R 5′-CCGTCAATTCMTTTRAGTTT-3.′ PCR reactions were performed in triplicate 20 μL mixture containing 4 μL of 5 × FastPfu Buffer, 2 μL of 2.5 mM dNTPs, 0.8 μL of each primer (5 μM), 0.4 μL of FastPfu Polymerase, and 10 ng of template DNA. Amplicons were extracted from 2% agarose gels and purified using an AxyPrep DNA Gel Extraction Kit (Axygen Biosciences, Union City, CA, U.S.) according to the manufacturer’s instructions. The original double-ended sequences were dehybridized using Trimmomatic software, and the average base quality was checked using the sliding window method. When the quality was <20, the previous high-quality sequence was discarded. The dehybridized bipartite sequences were spliced using the FLASH software. The splicing parameters were as follows: minimum overlap of 10 bp, maximum overlap of 200 bp, maximum mismatch rate, and maximum splicing rate of 1.5 bp. Chimeras of the FASTA sequences were removed from known databases using the UCHIME method for comparison. Unknown databases were removed using the self-comparison (*de novo*) method, and unsuitable short sequences were removed. Finally, the data were processed and analyzed using Trimmomatic (v 0. 36), and Vsearch (v 2. 7. 1) software.

### 2.7 Statistical analysis

The experimental data were organized using EXCEL 2010. One-way analysis of variance (ANOVA) was performed using SPSS20.0 statistical software, and multiple comparisons were performed using Tukey’s HSD method. Results were expressed as mean and standard error (mean ± SEM). A value of *p* < 0.05 was considered significant and 0.05 ≤ *p* < 0.10 as trends.

## 3 Results

### 3.1 Animal growth performance

Growth performance, including total weight gain, daily weight gain, and feed conversion ratio, is shown in Table 2. The differences in the initial weights and weaning ages of the calves in each group were not statistically significant (*p* > 0.05). Compared to the control group, weight gain in the AEOCO and AEO groups was significantly higher (*p* < 0.05). Furthermore, the feed conversion ratio was lower in the AEOCO group than that in the control group. There were no differences in the daily feed intake or dry matter intake between the treatment groups. The fecal scores were significantly higher in the AEOCO and AEO groups than in the control group. Dietary supplementation with different combinations of antibacterial agents positively affected the rate of diarrhea in calves. Among the treatment groups, AEO group showed the best effect, with the diarrhea rate reduced by 68.2% compared with that of the control group.

**Table 2:** Effects of different antibacterial compound combinations on growth performance and health in calves.

Item	CON	AEOCO	ACO	AEO	*p*-value
Initial weight (kg)	40.50 ± 1.22	39.67 ± 0.84	38.75 ± 1.54	39.08 ± 1.33	0.23
Weaning age (d)	66.17 ± 0.73	66.92 ± 0.58	66.42 ± 0.66	66.50 ± 0.50	0.15
Weight gain (kg)	44.75 ± 2.01^b^	52.75 ± 1.99^a^	47.08 ± 2.5^ab^	51.38 ± 2.91^a^	0.04
Average Daily Gain (g/d)	675.66 ± 27.48^a^	788.31 ± 28.53^b^	710.85 ± 39.64^ab^	771.81 ± 42.12^b^	0.03
Dry matter intake(g/d)	1199.13 ± 34.00	1223.06 ± 34.53	1191.56 ± 23.83	1276.88 ± 48.28	0.56
Feed conversion ratio	1.80 ± 0.07^a^	1.57 ± 0.07^b^	1.73 ± 0.09^ab^	1.71 ± 0.11^ab^	0.02
fecal score	2.65 ± 0.07^b^	2.82 ± 0.06^a^	2.76 ± 0.04^ab^	2.84 ± 0.07^a^	0.03
Diarrhea rate %	9.65%	4.39%	4.82%	3.07%	/

^a,b^Means in the same row with no common superscript letters differ significantly (*p* < 0.05).

### 3.2 Blood parameters

The effects of different combinations of antibacterial compounds on the serum total protein and antioxidant capacity of the calves are presented in Table 3. Total protein and globulin levels were lower in the AEO group than in the control group (*p* < 0.05). However, the albumin levels were higher in the AEOCO and AEO groups than in the control group (*p* < 0.05). No significant effect on antioxidant capacity was observed among the treatments.

**Table 3:** Effects of different antibacterial compound combinations on serum total protein and antioxidant capacity of calves.

Item	CON	AEOCO	ACO	AEO	*p*-value
Total protein(g/L)	61.32 ± 1.64^a^	60.71 ± 1.01^a^	58.94 ± 1.40^ab^	57.22 ± 0.78^b^	0.03
Albumin(g/L)	31.18 ± 1.12^b^	34.73 ± 0.62^a^	33.12 ± 0.94^ab^	34.21 ± 0.59^a^	0.04
Globulin(g/L)	30.22 ± 2.05^a^	25.98 ± 1.02^ab^	25.82 ± 1.29^ab^	23.01 ± 0.76^b^	0.04
T-AOC(mM)	0.45 ± 0.04	0.47 ± 0.03	0.45 ± 0.03	0.45 ± 0.03	0.26
GSH-PX(umol/L)	11.49 ± 2.21	15.72 ± 4.76	15.51 ± 2.94	11.21 ± 1.69	0.31
MDA(nmol/ml)	3.55 ± 0.21	4.40 ± 0.40	4.19 ± 0.50	4.02 ± 0.30	0.15

^a,b^Means in the same row with no common superscript letters differ significantly (*p* < 0.05).

### 3.3 Immune status

Serum immune indices of the calves are presented in Table 4. The levels were higher in the AEOCO group than in the control group (*p* < 0.05). The concentrations of IgG and IgM were higher in the AEOCO group than in the control group (*p* < 0.05). However, the concentration of IL-6 was lower in the AEOCO and AEO groups than that in the control group (*p* < 0.05). The concentration of TNF-α was decreased in ACO group (*p* < 0.05). No significant effect was observed on IFN-γ level.

**Table 4:** Effects of different antibacterial compound combinations on serum immune indices of calves.

Item	CON	AEOCO	ACO	AEO	*p*-value
IgA(ug/ml)	19.03 ± 0.42^b^	23.02 ± 0.61^a^	16.04 ± 0.27^c^	17.07 ± 0.40^c^	0.04
IgG(ug/ml)	207.28 ± 5.96^abc^	230.19 ± 7.60^a^	190.07 ± 3.98^d^	196.50 ± 5.44^cd^	0.03
IgM(ug/ml)	16.16 ± 0.49^b^	18.64 ± 0.56^a^	14.11 ± 0.32^c^	14.62 ± 0.39^c^	0.02
IL-6(pg/ml)	242.07 ± 4.79^b^	215.26 ± 3.88^c^	263.32 ± 4.56^a^	257.75 ± 4.33^a^	0.03
TNF-α(ng/L)	130.45 ± 5.00^bc^	110.32 ± 3.86^d^	145.20 ± 4.67^a^	140.58 ± 4.22^ab^	0.01
IFN-γ(pg/ml)	510.22 ± 16.67^ab^	470.05 ± 15.02^b^	540.85 ± 14.18^a^	530.37 ± 19.45^a^	0.03

^a,b,c,d^Means in the same row with no common superscript letters differ significantly (*p* < 0.05). IgA, immunoglobulin A; IgG, immunoglobulin G; IgM, immunoglobulin M; IL-6, interleukin-6; TNF-a, tumor necrosis factor.

### 3.4 Rumen fermentation

As shown in Table 5, there were no noticeable differences in ruminal fermentation between groups.

**Table 5:** Effects of different antibacterial compound combinations on rumen fermentation in calves.

Item	CON	AEOCO	ACO	AEO	*p*-value
pH	6.77 ± 0.15	6.80 ± 0.31	6.97 ± 0.18	6.77 ± 0.03	0.26
Total VFA (mmol/L)	60.19 ± 10.36	50.91 ± 12.09	45.68 ± 5.83	54.37 ± 2.99	0.35
Acetate	33.32 ± 5.24	29.03 ± 2.71	23.67 ± 3.13	26.62 ± 0.73	0.41
Propionate	19.42 ± 4.55	13.39 ± 6.69	12.92 ± 2.82	17.22 ± 2.58	0.22
Butyrate	4.72 ± 0.68	6.42 ± 4.12	6.61 ± 1.08	7.43 ± 1.77	0.56
Valerate	1.23 ± 0.39	0.76 ± 0.42	1.05 ± 0.08	1.66 ± 0.49	0.71
Isobutyrate	0.63 ± 0.21	0.57 ± 0.09	0.63 ± 0.12	0.62 ± 0.15	0.21
Isovalerate	0.85 ± 0.35	0.71 ± 0.16	0.80 ± 0.16	0.80 ± 0.15	0.15
Acetate/propionate(A:P)	1.79 ± 0.17	3.18 ± 1.03	1.96 ± 0.43	1.64 ± 0.32	0.19
NH_3_ -N	9.56 ± 0.87	12.17 ± 0.66	8.31 ± 0.35	9.16 ± 0.58	0.52
MCP	1.04 ± 0.23	1.14 ± 0.01	0.83 ± 0.19	0.88 ± 0.19	0.19

### 3.5 Ruminal bacteria community diversity and composition

The alpha diversity indices of the ruminal bacterial communities are shown in Table 6. The number of reads was higher in the ACO group than that in the control group (*p* < 0.05). The Chao 1 richness and ACE indices were higher in the AEOCO group than in the control group (*p* < 0.05).

**Table 6:** Effects of different antibacterial compound combinations on the microbial diversity index in rumen fluid of calves.

Item	CON	AEOCO	ACO	AEO	*p*-value
Reads	37,017 ± 4779^b^	46,933 ± 3567^ab^	51,555 ± 5190^a^	36,631 ± 3526^b^	0.03
Chao 1	814 ± 42^b^	1,195 ± 17^a^	1,169 ± 14^ab^	1,061 ± 94^ab^	0.02
Richness	632 ± 39^b^	1,039 ± 97^a^	985 ± 11^a^	861 ± 99^ab^	0.04
ACE	815 ± 36^b^	1,208 ± 18^a^	1,194 ± 14^ab^	1,082 ± 98^ab^	0.02

^a,b^Means in the same row with no common superscript letters differ significantly (*p* < 0.05).

**Figure 1:**
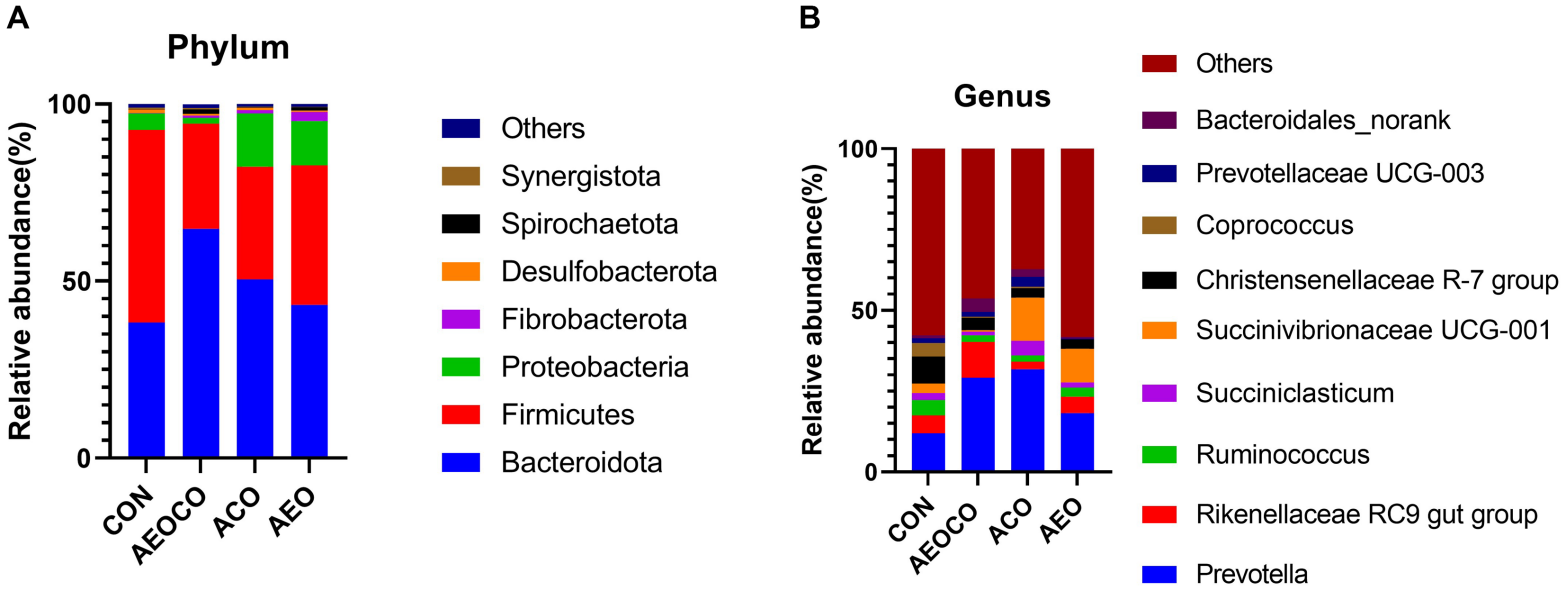
Effects of different antibacterial compound combinations on the rumen microbiome: **(A)** phylum composition of the microbiome **(B)** genus composition of the microbiome. CON group, basel diet; AEOCO group, attapulgite, plant essential oils, and chitosan oligosaccharide; ACO group, attapulgite and chitosan oligosaccharide; AEO group, attapulgite and plant essential oil.

At the phylum level (Figure 1A), seven bacterial phyla (Bacteroidetes, Firmicutes, Proteobacteria, Fibrobacter, Desulfobacterota, Spirochaetota, and Synergistota) were dominant with relatively high abundances (>1%). At the genus level (Figure 1B), *Precotella, Rikenekkaceae RC9 gut group, Ruminococcus, Succinlasticum, Succinivibrionaceae UCG-001,* and *Coprococcus* were dominant in all groups. As shown in Table 7, the ACO group had a significantly lower (*p* < 0.05) relative abundance of Firmicutes than the control group. The relative abundance of Bacteroidetes was the lowest in the control group, whereas those of Spirochaetota and Fibrobacteriota were the highest. The relative abundance of *Coprococcus* (*p* < 0.05) was lower in AEOCO group than in the control group. The relative abundances of *Succiniclasticum* and *Bacteroidales_norank* were higher in the ACO and AEO groups (*p* < 0.05).

**Table 7:** Main microbiome (accounting for ≥0.5% of the total sequences in at least one of the samples) that significantly changed among different treatments.

Item	CON	AEOCO	ACO	AEO	*p-*value
Phylum					
Firmicutes	39.35 ± 11.2^b^	44.69 ± 4.35^ab^	27.89 ± 2.25^a^	31.86 ± 12.21^ab^	0.02
Bacteroidota	43.28 ± 4.26^b^	48.36 ± 3.36^a^	54.44 ± 3.12^a^	50.49 ± 1.15^a^	0.03
Spirochaetota	0.92 ± 0.02^a^	0.21 ± 0.05^b^	0.53 ± 0.03^ab^	0.22 ± 0.01^b^	0.04
Fibrobacteota	2.61 ± 1.06^a^	0.03 ± 0.01^b^	0.63 ± 0.01^b^	1.04 ± 0.01^ab^	0.02
Genus					
*Coprococcus*	0.09 ± 0.01^b^	2.45 ± 1.02^a^	1.5 ± 0.01^ab^	0.38 ± 0.01^b^	0.01
*Succiniclasticum*	1.63 ± 1.02^b^	1.82 ± 1.06^b^	4.27 ± 1.06^a^	4.54 ± 2.02^a^	0.04
*Bacteroidales_norank*	0.51 ± 0.02^b^	1.19 ± 1.01^ab^	1.68 ± 1.02^a^	2.35 ± 1.06^a^	0.03

^a,b^Means in the same row with no common superscript letters differ significantly (*p* < 0.05).

## 4 Discussion

This study examined the effects of different combinations of antibacterial additives (attapulgite, plant essential oils, and chitosan oligosaccharides) on the growth, health, and rumen microbiome of calves. Current research indicates that supplementation with attapulgite significantly reduces the rate of diarrhea and improves the growth performance of calves (14). The addition of plant essential oils to feed can enhance the immune system and antioxidant capacity of calves (29). The addition of 5 g of chitosan oligosaccharides per day to the milk replacement of calves increased their average daily weight gain and reduced the incidence of diarrhea (30). We found that the simultaneous addition of attapulgite, plant essential oils, and oligochitosan significantly increased the daily weight gain with the strongest enhancement effect, and the feed-to-weight ratio was significantly reduced by 12.78%. Addition of essential oils or oligochitosan alone was not as effective as combined additions for increased calf growth performance. The results also showed that essential oils and oligochitosan improved the feed intake and daily weight gain of calves, and the effects could be combined, which is consistent with the results of previous studies (14, 31). The addition of different antimicrobial combinations improved the fecal score, which was related to the ability of attapulgite to adsorb toxins and protect the mucous membranes of the gastrointestinal tract. The addition of attapulgite and essential oils also significantly reduced the calf diarrhea rate by more than 50%. The best reduction was achieved in ACO group without the addition of oligochitosan, suggesting an antagonistic effect between oligochitosan and other antimicrobial materials for the optimization of calf diarrhea.

Serum biochemical indicators reflect metabolic function and functional changes in related tissues and organs. The total protein in serum includes albumin and globulin. This directly reflects the level of protein digestion and absorption in the animals (32). Serum immunoglobulin (Ig) levels are important indicators of humoral immunity and play an important role in improving autoimmunity (33). IgA, IgM, and IgG are secreted by plasma cells and are the main immunoglobulins in animals that resist germ infections and diseases. The concentration of immunoglobulins in the serum directly reflects the strength of calf immunity (34, 35). IL-6 and TNF-α secreted by monocyte macrophages and Th1 cells are direct mediators of stress injury and promote inflammation (36). In this study, the addition of different antimicrobial combinations increased serum albumin concentration. Moreover, the combination of attapulgite, oligochitosan, and essential oils significantly increased the serum IgA, IgG, and IgM concentrations, whereas the other two combinations decreased them, indicating that the antimicrobial material combinations could play a better role in improving the immunity of calves only when the three kinds of antimicrobial materials were present. The lack of essential oils or oligochitosan in the combinations weakened immunity to varying degrees. In addition, the combination of attapulgite, oligochitosan, and essential oils significantly reduced the serum levels of IL-6, TNF-α, and IFN-γ while the other two combinations increased the serum levels of IL-6, TNF-α, and IFN-γ. This suggests that simultaneous use of these three antimicrobial agents can reduce inflammatory reactions in calves and improve their health. Published research has indicated that in sow growth performance tests, the stillbirth rate of the plant essential oil applied group was reduced by 53.33% compared to that of the antibiotic applied group. Moreover, plant essential oils significantly increased the immunoglobulin content of sow serum and improved the survival rate of weaned piglets (37). The levels of cytokines and serum immunoglobulins in piglets were also found to decrease during weaning stress. However, the addition of oligochitosan to the diet decreased the piglets in weaning stress cytokines and serum immunoglobulins, enhanced the expression of the *IL-1β* gene *in vivo*, and increased the serum levels of immunoglobulins (IgM, IgA, and IgG) and cytokines (IL-2, IL-1β, and IL-6), which is in agreement with our findings (23).

Rumen microbial balance plays an important role in host defense mechanisms in ruminants. In the present study, all combinations of antimicrobial agents increased the abundance of calf ruminal flora and promoted ruminal health. The combination of attapulgite, oligochitosan, and plant essential oils was most effective in enhancing the abundance of rumen flora in calves. In a study on laying hens, the intestinal flora structure was improved by increasing the number of beneficial bacteria in the cecum through the addition of attapulgite to the diet (38), which is consistent with the results of our study. Bacteroidetes and Firmicutes were the dominant phyla in the rumen, suggesting that they play important roles in ruminal growth and development (39). The dominant phyla in the calves of each group in this study were Firmicutes, Bacteroidetes, and Proteobacteria, consistent with those reported in previous studies. Firmicutes play a role in the energy utilization of calf organisms, and a high abundance of Firmicutes ensures energy supply during the period of rapid calf development. Bacteroidetes are mainly responsible for the degradation of carbohydrates in the rumen and the hydrolysis of proteins (40). The addition of attapulgite, oligochitosan, and plant essential oils increased the abundance of Bacteroidetes. Our results indicate that attapulgite, oligochitosan, and plant essential oils would enhance the digestion and absorption of proteins and carbohydrates. In addition, *Succiniclasticum* was highly abundant in all the three experimental groups. *Succiniclasticum*, the main bacterium producing propionic acid (the main precursor of gluconeogenesis), shows a significant positive correlation with animal feed efficiency and abundance (41).

Our study had several limitations. First, the number of calves selected was small. Our conclusions would be more reliable if the sample size was larger. Second, the current study did not fully consider the possibilities of all antimicrobial material combinations. More combinations of different antimicrobial materials, such as combinations of oligochitosan and essential oils, could be designed in the future. Third, this study did not compare the microbial composition of calf feces between groups. Further studies can examine the possibility that antimicrobial material combinations have inhibitory effects on pathogenic bacteria that cause diarrhea in calves.

## 5 Conclusion

The addition of attapulgite, plant essential oils, and oligochitosan to feed can improve calf growth performance, specifically by increasing the daily weight gain and feed intake of Holstein calves before weaning. It can reduce calf diarrhea rates by increasing serum immunoglobulin concentrations and decreasing serum inflammatory factor concentrations. Furthermore, the addition of attapulgite, plant essential oils, and oligochitosan can increase the abundance of ruminal microorganisms. The most effective results were obtained with the combined addition of attapulgite, plant essential oils, and oligochitosan.

## Data availability statement

The datasets presented in this study can be found in online repositories. The names of the repository/repositories and accession number(s) can be found in the article/supplementary material.

## Ethics statement

The animal studies were approved by the experimental design and procedures were performed in accordance with the Institutional Animal Care and Use Committee of Nanjing Agricultural University, China (SYXK2011-0036). The studies were conducted in accordance with the local legislation and institutional requirements. Written informed consent was obtained from the owners for the participation of their animals in this study.

## Author contributions

SL: Conceptualization, Formal analysis, Methodology, Writing – original draft, Writing – review & editing. CW: Investigation, Conceptualization, Methodology, Writing – original draft, Writing – review & editing. HZ: Writing – original draft, Writing – review & editing. ZH: Funding acquisition, Project administration, Resources, Validation, Visualization, Writing – original draft, Writing – review & editing.
